# Bacteriological aspects of chronic osteoarticular infections in adults: the influence of the osteosynthesis material

**DOI:** 10.1186/s13104-017-2976-z

**Published:** 2017-11-28

**Authors:** Adil Maleb, Mohammed Frikh, Yassine Ben Lahlou, Belkacem Chagar, Abdelhay Lemnouer, Mostafa Elouennass

**Affiliations:** 1 Bacteriology Department, Mohammed V Military Teaching Hospital, Rabat, Morocco; 2 Research Team: Bacterial Epidemiology and Resistance, Mohammed V University, Faculty of Medicine and Pharmacy, Rabat, Morocco; 3 Traumalogy Department, Mohammed V Military Teaching Hospital, Rabat, Morocco

**Keywords:** Antibiotic resistance, Bacteria, Infectious diseases, Orthopedic surgery

## Abstract

**Background:**

The aim of this study is to establish the bacterial epidemiology of chronic osteoarticular infections in adults, to study the susceptibility of the isolated strains to antibiotics and to demonstrate the influence of osteosynthesis material thereon.

**Patients and methods:**

This is a retrospective study of 78 months, from January 2006 to June 2012, providing bacteriological samples from patients with osteitis and osteoarthritis in the Mohammed V military teaching hospital of Rabat. Isolation and identification of bacteria were made by bacteriological classical techniques. The antimicrobial susceptibility testing of the isolates was performed by disk diffusion agar method, as recommended by the Committee of the susceptibility of the French Society for Microbiology (CA-SFM).

**Results:**

We collected 234 cases, 53% (n = 124) of patients without osteosynthesis material (group A) and 47% (n = 110) patients with osteosynthesis material (group B).We isolated 371 bacteria which 51.49 (n = 191) in group A and 48.51% (n = 180) in group B. Gram-positive cocci were the most frequent (n = 234), followed by the Gram-negative bacilli (n = 114) and the Gram-positive bacilli (n = 19). Our study shows that the rate of resistance to antibiotics in strains obtained from patients with osteosynthesis material is higher compared to those obtained from patients without osteosynthesis material.

**Conclusions:**

Chronic OA infection in adults is difficult to diagnose and treat. Its good management must be multidisciplinary.

## Background

The support of osteoarticular (OA) infections is often a difficult challenge. Usually chronic forms are not life threatening. However, they expose patients to serious disabling situations such as prolonged hospitalization, high total disability and inability up to 1 year. Furthermore, osteoarticular infections are a major therapeutic problem due to the selection of resistant mutants [1–3].

The empirical antibiotic therapy has no place in OA infections, except in some emergency cases  [4]. The optimal treatment of these infections; with or without osteosynthesis material (metal implants), passes first by a bacteriological documentation and a study of the sensitivity of the relevant bacteria to antibiotics [5].

The aim of this study is to establish the bacterial epidemiology of chronic osteoarticular infections in adults, to study the susceptibility of the isolated strains to antibiotics and to demonstrate the influence of osteosynthesis material thereon.

## Patients and methods

This is a retrospective study of 78 months, from January 2006 to June 2012, providing bacteriological samples from patients with osteitis and osteoarthritis in the Mohammed V military teaching hospital of Rabat. Bacteriological samples were distributed into two groups, group A: patients without osteosynthesis material and group B: patients with osteosynthesis material (rods, plates and screws). They were obtained by aspiration of the adjacent liquid of osteitis site during surgical exploration, by sampling tissue, or bone fragments, deep trimming product during surgery, or aspiration of joint fluid. Removed osteosynthesis material were sonicated, and solid samples were crushed in order to destroy the adherent biofilm and detach the bacteria to facilitate culture. All samples were inoculated under a microbiological safety cabinet type II, by a technician wearing sterile gloves to avoid contamination. Whether liquid or solid, all samples were inoculated onto rich media usually used in the laboratory: blood agar incubated in aerobic and anaerobic conditions at 37 °C, chocolate agar supplemented with multivitamins in 5% of CO2 at 37° C, traditional aerobic and anaerobic blood culture broths. Incubation of culture media were prolonged (at least 7 days) to allow the growth of fastidious bacteria, slow growing. Cultures under aerobic and CO2 were observed daily, anaerobic cultures every 48 h, the enrichment broths were transplanted as soon as a disorder or a culture is observed; they were systematically replanted after at least 7 days of incubation on agar culture media and incubated again additional 48 h. Culture of Mycobacteria was not routinely performed except if the clinician specifically requests it. Identification of bacteria were made by bacteriological classical techniques. The antimicrobial susceptibility testing of the isolates was performed by disk diffusion agar method, as recommended by the Committee of the susceptibility of the French Society for Microbiology (CA-SFM) [6]. Carbapenemases were sought by the test of Hodge (TH) in accordance with the same previous recommendations. The duplicates were collected only once and the bacteria belonging to the commensal flora (*Coagulase negative Staphylococci*, *Corynebacterium* spp., etc.) have been retained only when the same antibiotype was isolated many times.

## Results

During this period of study we collected 234 samples (Table 1) originating from 234 different patients, 53% (n = 124) of group A and 47% of group B (n = 110). In each case we isolated at least one bacterium. The Table 2 shows that culture permitted to isolate 371 bacteria which 51.49 (n = 191) in group A and 48.51% (n = 180) in group B. Among isolates, Gram-positive cocci were the most frequent (n = 234), followed by the *Gram*-*negative bacilli* (n = 114) and the *Gram*-*positive bacilli* (n = 19). Among only 34 specific types of research of mycobacteria, we isolated four strains of *Mycobacterium tuberculosis*, all in group A. The difference was in the distribution of species between these two groups. With some exceptions, we found that the cocci predominated in group A and bacilli predominated in group B (Table 2).

**Table 1:** Distribution of specimens type (n = 234)

	Group A (n = 124)	Group B (n = 110)	Group A + B (n = 234)
Deep pus	46	50	96
Bone	53	33	86
Joint fluid	7	11	18
Soft tissue	8	5	13
Fistulaswab	10	2	12
Material	0	9	9

**Table 2:** Distribution of isolates by groups and species (n = 371)

	Group A	Group B
	Patients without osteosynthesis material (n = 191)	Patients with osteosynthesis material (n = 180)
*Gram*-*positive cocci*		
*Staphylococci*		
*Coagulase*-*negative Staphylococci*	41	47
*Staphylococcus aureus*	53	32
*Streptococci*		
*Streptococcus* spp.	14	5
*β*-*hemolytic streptococci*	7	3
*Enterococci*		
*Enterococcus faecalis*	14	9
*Enterococcus faecium*	1	4
Others		
*Micrococcus* spp.	0	2
*Leuconostoc* spp.	2	0
*Gram*-*negative bacilli*		
*Enterobacteriaceae*		
*Klebsiella pneumoniae*	6	12
*Enterobacter cloacae*	6	12
*Escherichia coli*	8	7
*Proteus mirabilis*	2	5
*Klebsiella oxytoca*	0	3
*Morganella morganii*	2	1
*Citrobacter koseri*	2	0
*Citrobacter freundii*	1	1
*Salmonella typhimurium*	2	0
*Proteus vulgaris*	0	1
*Non*-*fermenting bacteria*		
*Pseudomonas aeruginosa*	11	12
*Acinetobacter baumannii*	5	4
*Pseudomonas mendocina*	0	2
*Ochrobactrum anthropi*	0	2
*Ralstonia pickettii*	0	1
*Burkholderia cepacia*	0	1
Others		
*Aeromonas hydrophila*	1	4
*Gram*-*positive bacilli*		
*Corynebacterium* spp.	7	9
*Bacillus* spp.	1	0
*Listeria monocytogenes*	0	1
*Actinomyces* spp.	1	0
*Acid*-*fast bacilli* (of 34 requests)		
*Mycobacterium tuberculosis*	4	0

**Fig. 1:**
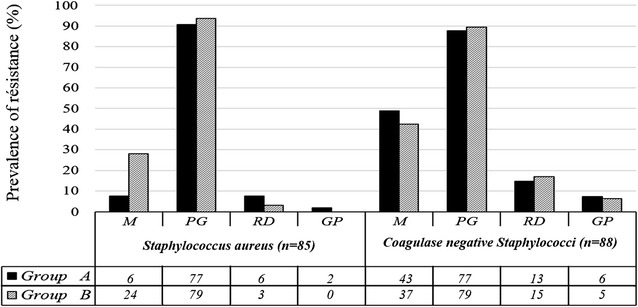
Level of resistance of isolated *Staphylococci*. *M* methicillin, *PG* penicillin G, *RD* rifampicin, *GP* glycopeptides

The *Staphylococci* resistance rates of group A and group B are shown in Fig. 1. The rate of resistance of streptococci to penicillin G was 19.5% (n = 4) in group A and 37.5% (n = 3) in group B. The *Enterococci* resistance rates to ampicillin were none in group A and 15.38% (n = 2) in group B. The resistance rates of *Streptococci* and *Enterococci* to rifampicin was 5.56% (n = 2) in group A and 4.76% (n = 1) in group B.

Regarding the *Enterobacteriaceae*, the percentage of strains producing extended spectrum beta-lactamases with was 13.79% (n = 4) in group A and 42.86% (n = 18) in group B. One strain was producing carbapenemase, it belonged to group B. The overall rate of *Gram*-*negative bacilli* resistance to ciprofloxacin was 18.6% (n = 16) in group A and 42.65% (n = 29) in group B.

## Discussion

In our series, we acknowledge that the study was only descriptive and no statistical analyses were performed and that many of the n = numbers for microorganism species were too small to analyse with confidence.

*Gram*-*positive cocci* were isolated in approximately two-thirds of patients (n = 234; 63.07%) and Gram-negative bacilli in less than one in three (n = 114; 30.72%) [In the distribution of the isolates of the total number of collected cases (A + B)]. The remaining isolates were represented by *Gram*-*positive bacilli* (n = 19) and *Mycobacteria*. Our series shows, as for other studies, that *S. aureus* (n = 85, 22.90%) was the most frequent causative agent regardless of the osteitis’s type [1]. *Staphylococci* together accounted for only 46.63% (n = 173) of all isolates. Their role in bone infections is related to their presence at the cutaneous and mucosal level, their adaptation to infection of the bone (they adhere easily to the bone, cartilage, and surgical implants) [1, 7]. *Streptococci* and *Enterococci* were poorly isolated in our series. This would be partly due to the fact that they are frequently found during early infection. Indeed, they are rarely identified in chronic OA infections, due to their good response to antibiotics [8].

The *Gram*-*negative bacilli* were mainly represented by *K. pneumoniae*, *E. cloacae*, *E. coli*, and *P. mirabilis* in the group of *Enterobacteriaceae* and *P. aeruginosa* and *A. baumannii* in the *non*-*fermenting Gram*-*negative bacilli* group. These bacteria are more closely related to a nosocomial superinfection of the implanted osteosynthesis material, in some cases and for some species they may be secondary to post-traumatic osteitis [1, 8]. *Gram*-*positive bacilli* were dominated in our series by *Corynebacteria*. This group was generally considered contaminants but is currently increasing and described as the one responsible for osteitis. These species, such as *coagulase*-*negative Staphylococci*, originate from the skin or the environment and often dominate in intraoperative infections. They present problems of interpretation; the responsibility can be accepted only on deep samples. In these cases, isolation of the same organism in at least two locations or two samples is very comforting, justifying the increase in sampling sites [6].

In our series, the distribution of isolates between the two groups A and B shows that the *cocci* predominated in group A, while bacilli predominated in group B. The distribution by species shows that some bacteria derogate the distribution by groups. These include a predominance of *E. coli*, *M. morganii*, *Citrobacter* spp., *S. typhimurium*, and *A. baumannii* in group A and a slight predominance of *coagulase*-*negative staphylococcal species* in group B. The isolation of *Enterobacteriaceae* and *A. baumannii* in group A in our series would be linked to their community origin, despite their multiple resistances to antibiotics which is often seen in nosocomial strains [9–11].

The results of our study show that the rate of resistance to antibiotics in strains obtained from patients with osteosynthesis material are higher compared to those obtained from patients without osteosynthesis material. This is usually about multiresistant bacteria. Especially: Methicillin-resistant *Staphylococcus aureus* (MRSA), *Streptococci* resistant to penicillin G, *Enterococci*resistant to ampicillin, *Enterobacteriaceae* producing extended spectrum beta-lactamases (ESBLs), *Enterobacteriaceae* producing carbapenemase and *Gram*-*negative bacilli* resistant to ciprofloxacin.

Antibiotic resistance is an additional difficulty that complicates the therapeutic treatment of OA infections on the osteosynthesis material. This resistance is acquired by the bacteria before and/or after contamination of the patient subject to OA infection. Before contamination, the nature of nosocomial OA infections explains very clearly the transmission of multiresistant bacteria immediately among patients. After contamination, a multitude of factors contributes to the resistant mutant selection [1, 12, 13]:

- Unreflective use of antibiotics.
- High efficiency of bone antibiotics are also more involved in bacterial resistance selection (rifampicin, fosfomycin, fluoroquinolones, clindamycin, fusidic acid).
- Deep localization of the OA infection significantly limits the spread of antibiotics.
- Osteosynthesis material causes the formation of biofilm, with all the consequences: limiting the spread of antibiotics, loss of sensitivity to certain antibiotics, prolonged persistence in osteoblasts, escape the immune defense mechanism.

## Conclusion

Chronic OA infection in adults is difficult to diagnose and treat. Patients who have had orthopedic implants, when they become infected, are more likely to be infected with bacteria more virulent, more resistant to antibiotics, and better escape the immune system.
